# Effect of Aldose Reductase Inhibition on Interleukin-1β-Induced Nitric Oxide (NO) Synthesis in Vascular Tissue

**DOI:** 10.1080/15604280212523

**Published:** 2002

**Authors:** Juan Morales, Joseph C. Dunbar, Jeffrey L. Ram

**Affiliations:** 1 Department of Physiology Wayne State University School of Medicine 540 E. Canfield Detroit MI 48201-1928 USA

## Abstract

Glucose metabolism via sorbitol pathway has been implicated as a possible contributor to the diabetes-related vascular changes. Nitric oxide plays a major regulatory role in the vascular dilatatory and constricted response. Also it has been observed that diabetes causes vascular changes leading to a decrease in nitric oxide production. Additionally the accumulation of sorbitol is also related to decreased nitric oxide production. In the present study we investigated the effect of normal and high glucose in the presence or absence of both interleukin-1β or an aldose reductase inhibitor on nitric oxide production in rat aortic rings *in vitro*. Aortic rings from normal male Wistar rats were dissected and incubated for 24 to 48 hrs in the presence of glucose (5.0 mM or 20 mM) or with or without interleukin (20 ng/ml). Other rings were incubated in the above media with the addition of the aldose reductase inhibitor (WAY 121509). Interleukin-1β stimulated the 24 hr nitric oxide production and WAY 121509 decreased it under both low and high glucose culture conditions. The interleukin-1β stimulation was continued for 72 hrs. Nitric oxide production in response to interleukin-1β was greater at all time points when compared to the incubation in media without interleukin-1β. In media containing WAY 121509 the nitric oxide production was decreased. Interleukin-1β stimulated a greater increase in nitric oxide production from aortic rings when incubated in high glucose when compared to normal glucose. The inhibitory effect of aldose reductase inhibition was reversible after 24 hr inhibition under both normal and high glucose conditions. We conclude that high glucose enhances the interleukin- 1β-induced nitric oxide synthesis and the cytokine-induced nitric oxide production was inhibited by aldose reductase inhibition. Nitric oxide production may be linked to redox influences caused by the polyol pathway.


*Int. Jnl. Experimental Diab. Res.*, 3:11-20, 2002
© 2002 Taylor and Francis
1560-4284/02 $12.00 + .00

### Effect of Aldose Reductase Inhibition on Interleukin-1ß-induced Nitric Oxide (NO) Synthesis in Vascular Tissue

JUAN MORALES, JOSEPH C. DUNBAR* AND JEFFREY L. RAM

Department of Physiology, Wayne State University, School of Medicine, 540 E. Canfield, Detroit, MI 48201-1928, USA

*Received: June 28, 2001; In final form:November 13, 2001*

**Glucose metabolism via sorbitol pathway has been implicated as a possible contributor to the diabetes-related vascular changes. Nitric oxide plays a major regulatory role in the vascular dilatatory and constricted response. Also it has been observed that diabetes causes vascular changes leading to a decrease in nitric oxide production. Additionally the accumulation of sorbitol is also related to decreased nitric oxide production. In the present study we investigated the effect of normal and high glucose in the presence or absence of both interleukin-1β or an aldose reductase inhibitor on nitric oxide production in rat aortic rings *in vitro*. Aortic rings from normal male Wistar rats were dissected and incubated for 24 to 48 hrs in the presence of glucose (5.0 mM or 20 mM) or with or without interleukin (20 ng/ml). Other rings were incubated in the above media with the addition of the aldose reductase inhibitor (WAY 121509). Interleukin-1β stimulated the 24 hr nitric oxide production and WAY 121509 decreased it under both low and high glucose culture conditions. The interleukin-1β stimulation was continued for 72 hrs. Nitric oxide production in response to interleukin-1β was greater at all time points when compared to the incubation in media without interleukin-1β. In media containing WAY 121509 the nitric oxide production was decreased. Interleukin-1β stimulated a greater increase in nitric oxide production from aortic rings when incubated in high**

*Corresponding author: tel: (313) 577-1520; fax: (313) 577-5494; e-mail: jdunbar@med.wayne.edu

**glucose when compared to normal glucose. The inhibitory effect of aldose reductase inhibition was reversible after 24 hr inhibition under both normal and high glucose conditions. We conclude that high glucose enhances the interleukin-1β-induced nitric oxide synthesis and the cytokine-induced nitric oxide production was inhibited by aldose reductase inhibition. Nitric oxide production may be linked to redox influences caused by the polyol pathway.**

### INTRODUCTION

The diversion of glucose into the sorbitol pathway has been extensively studied as one of the possible mechanisms leading to diabetes-associated-disease in the eyes, kidneys, nerves, and blood vessels [1-3]. Specific aspects of the sorbitol pathway producing physiological changes that might lead to dysfunction include the accumulation of polyols, inositol depletion, NADPH depletion, and oxidative stress, which alter osmolarity, signal transduction, activity of NADPH-dependent enzymes, and cell biochemistry [4, 5]. This augmentation of the sorbitol pathway has been demonstrated particularly in blood vessels and vascular cells [6, 7].

Nitric oxide synthesis and activity plays a major regulatory role in the regulation of vascular dilation and contraction. The dysfunction of nitric oxide synthase and/or decrease in the nitric oxide production system has also been suggested as a major physiological alteration in diabetes [8-10].

Many studies have investigated the effects of inhibiting aldose reductase on the pathologies of diabetes [11-13]. In animal models, aldose reductase inhibitors (ARIs) have been effective in preventing the pathology (nephropathy, neuropathy and retinopathy) associated with experimental diabetes [12-14]. Furthermore, ARIs have been shown to reduce some of the biochemical markers (polyol accumulation, inositol depletion) associated with diabetes in cell culture systems [15-18]. One interesting aspect of the ARI effect is that they are most effective at reducing pathology during the onset of diabetes but seem less effective in reversing the pathology and its progression after it has been induced [19-22].

Several reports indicate that ARIs can be used to test, in part, the hypothesis that the sorbitol pathway mediates effects of high glucose on isolated tissues [16, 22]. There are also reports that metabolism through the sorbitol pathway leads to a decrease in nitric oxide synthase due to the depletion of NADPH [23]. In this study we investigated the effects of aldose reductase inhibition (ARI WAY 121509) on interleukin-1β-induced increased nitric oxide synthesis in rat aortic rings in the presence of low and high glucose concentrations.

### MATERIALS AND METHODS

Normal male Wistar rats (Harlan Laboratories) were used. Animals were maintained on standard laboratory chow and water. The protocol for the study was approved by the Animal Investigation Committee. The rats were killed by decapitation and aortic rings were dissected and incubated for 24 h in minimal essential media containing glucose 5.0 mM (NG) or glucose containing 20 mM (HG). Other media contained NG plus interleukin (20 ng/ml; NG-IL) or high glucose plus interleukin (20 ng/ml; HL-IL). In addition, other rings were incubated in the above media plus 0.05% dimethylsulfoxide (0.05% DMSO, vehicle) or 100 mg/ml of the aldose reductase inhibitor, WAY 121509. The concentration of WAY 121509 used in these experiments was calculated based on the range of concentrations found in plasma of diabetic or galactosemic animals treated with this drug [24-27].

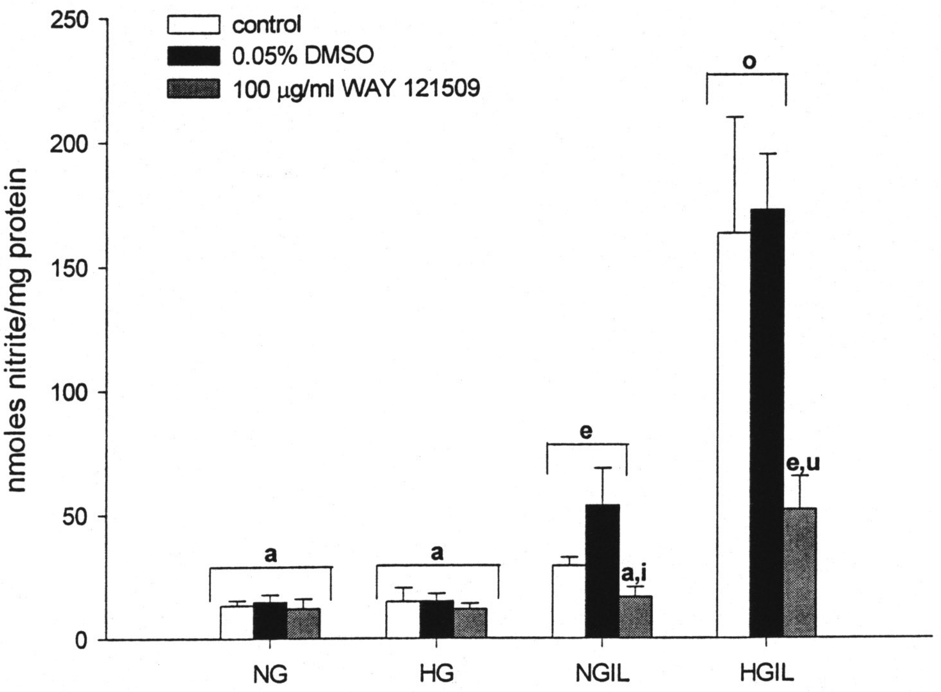

FIGURE 1
Effect of WAY 121509 (100 mg/ml) on the interleukin-1β-stimulated NO production in rat aortic rings incubated for 24 h under normal and high glucose conditions. **Open bars**: defined as control treatments (NG, HG, NGIL, and HGIL). **Filled bars**: rings in the same four treatments plus the addition of the vehicle 0.05% DMSO. **Cross-hatch bars**: same four treatments plus the aldose reductase inhibitor WAY 121509 and 0.05% DMSO. NO estimation was performed via nitrite/nitrate measurement by the enzymatic/Griess reaction method. ANOVA repeated measures (Student-Newman-Keuls Method), $p<0.001$. Different vowels represent statistical difference among groups at $p<0.05$. N= 6 animals.

### *MULTI-DAY EXPERIMENTS*

In other experiments, rat aortic rings were incubated in the different media as described above, but were maintained for three periods of 24 h. Every 24 h the cell media were replaced with media identical to the treatment in which the aortic rings had been incubated. The objective of this experiment was to study the time course of NO synthesis by aortic rings in each of the treatments when maintained the same after 24 h media, with replacement during the 72 h paradigm. The data collected provides a baseline for comparison of the effects of replacing media with non-identical treatments after the initial 24 h period.

In the same experiments, some rings were selected for studying the effect of replacement with media containing 0.05% DMSO/interleukin-1β or media containing 100 mg/ml WAY 121509 plus interleukin-1β and viceversa after the initial 24 h period. These experiments evaluated the effect of WAY 121509 on NO synthesis already induced by interleukin-1β in aortic rings incubated with normal and high glucose media. In addition, these experiments determined whether removal of WAY 121509 after the initial 24 h period had long lasting effects on NO synthesis.

### *NO ANALYSIS*

The NO synthesis measurements were analyzed via enzymatic/Griess reaction method.

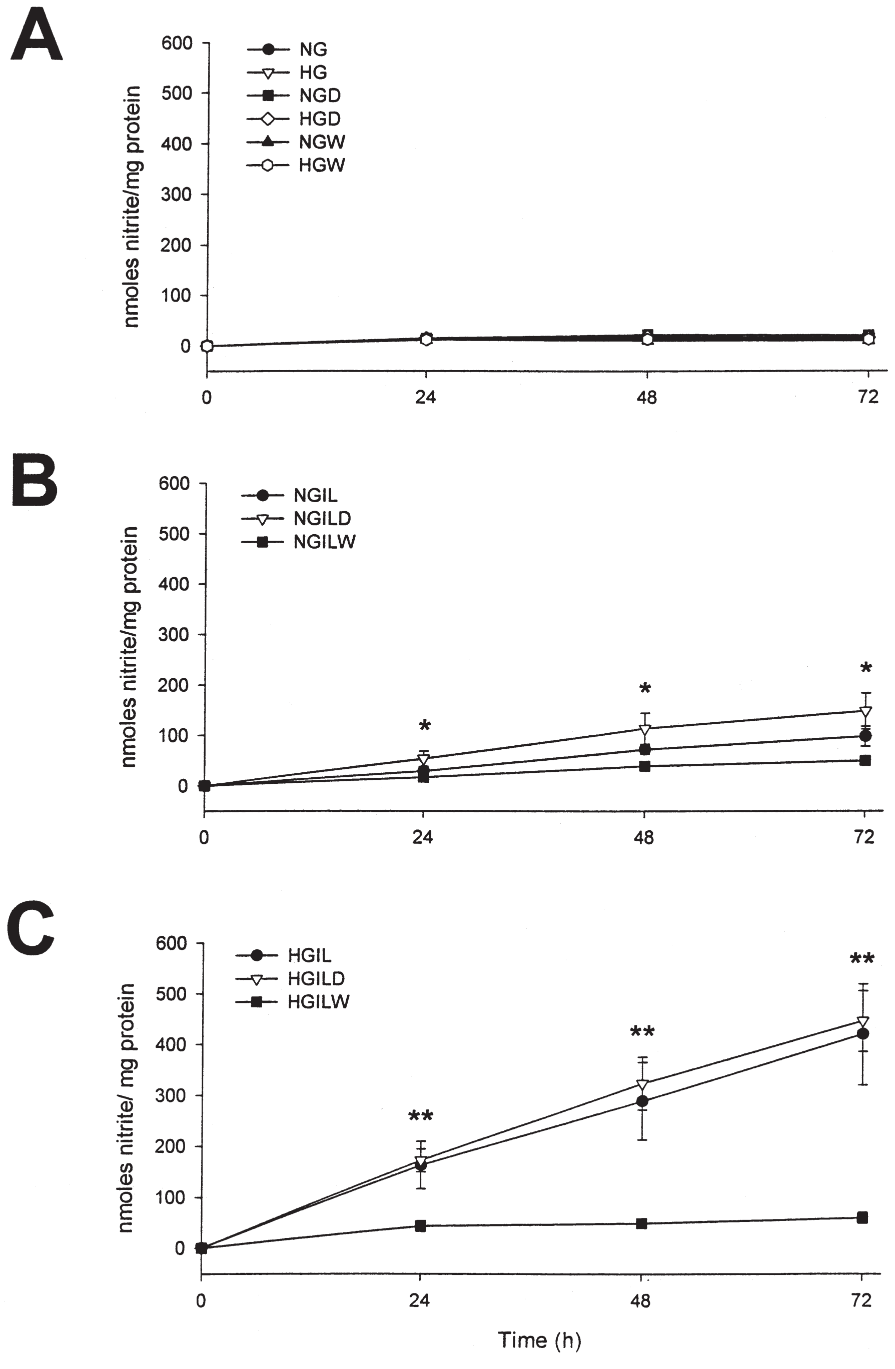

FIGURE 2
A 72 h study showing the time course for NO synthesis in twelve different groups of rat aortic rings. Panel A: Aortic rings incubated in interleukin-1β-free treatments (NG, HG, NGD, HGD, NGW, and HGW); Panel B: Aortic rings incubated in normal glucose media containing interleukin-1β only or in combination with either vehicle (0.05% DMSO) or 100 mg/ml WAY 121509; Panel C: Aortic rings incubated in high glucose media containing interleukin-1β only or in combination with either vehicle (0.05% DMSO) or 100 mg/ml WAY 121509. ANOVA repeated measures (Student-Newman-Keuls Method), $p<0.001$; *, NGILD versus NGILW at all time points and NGILD and NGIL versus treatments in Panel A, $p<0.05$; **, HGILD and HGIL versus HGILW at all time points, HGILD and HGIL versus treatments in Panels A and B at all time points, $p<0.05$. N= 6 animals.

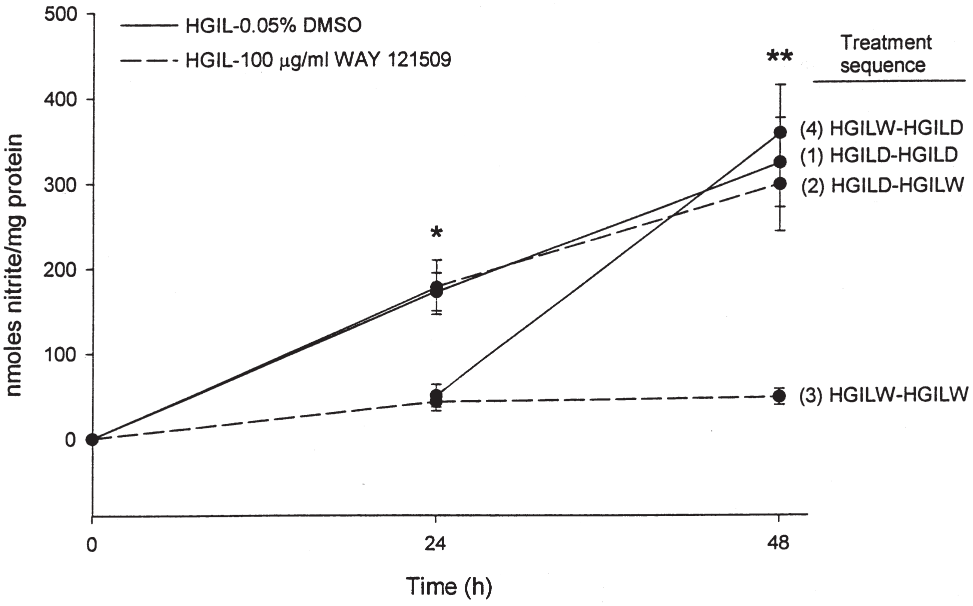

FIGURE 3
Effect of WAY 121509 (100 mg/ml) on the interleukin-1β-induced NO synthesis in rat aortic rings during 48 h incubations under high glucose conditions. Media was sampled and replaced at the end of 24 h and sampled at the end of the next 24 h (time point= 48 h). **Solid lines** represent periods during which rings were incubated in the presence of 0.05% DMSO. **Dashed lines** represent periods during which rings were incubated in the presence of WAY 121509 in 0.05% DMSO. Curve 1: rings treated with HGIL containing 0.05% DMSO during the first 24 h incubation period and replaced with identical treatment for the second 24 h. Curve 2: rings treated with HGIL containing 0.05% DMSO during the initial 24 h period and replaced with HGIL containing 100 mg/ml WAY 121509 during the second 24 h. Curve 3: rings treated with HGIL containing 100 mg/ml WAY 121509 during the first 24 h incubation period and replaced with identical treatment for the second 24 h. Curve 4: rings treated with HGIL containing 100 mg/ml WAY 121509 during the initial 24 h period and replaced with HGIL media containing 0.05% DMSO during the last 24 h period. ANOVA repeated measures (Student-Newman-Keuls Method), $p<0.001$; *, incubation sequence (1, 2) versus (3, 4) at 24 h; and **, incubation sequence (3) versus (1, 2, and 4) at 48 hr, $p<0.05$. N= 6 animals.

Data was analyzed using ANOVA with repeated measures with post-hoc analysis using Student-Newman-Keuls.

### RESULTS

Aortic rings incubated with 5 mM (NG) and 20 mM (HG) glucose in interleukin-1β-free media contained similar nitrate/nitrite levels compared to rings in identical media containing 100 mg/ml WAY 121509 or vehicle (0.05% DMSO). Interleukin-1β significantly induced nitric oxide (NO) production and WAY 121509 (100 mg/ml) attenuated by 60% this response in aortic rings incubated for 24 h with either normal or high glucose media (Figure 1). Vehicle (0.05% DMSO) did not interfere with the interleukin-1β-induced NO production.

The multi-day experiments, in which the media were replaced every 24 h but the same treatment was present for 72 hr, confirmed a 24 hr stimulation that was continued for 72 hr (Figure 2). These experiments further demonstrated that for the treatments that induced an increase in NO synthesis, the synthesis of NO increased linearly from day to day. Treatments containing interleukin-1β in the absence of

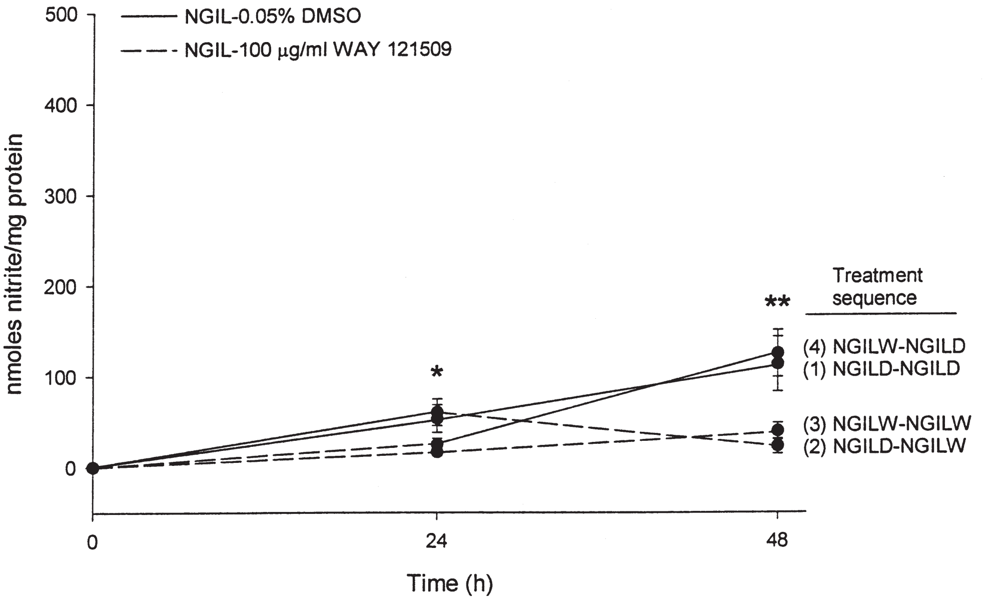

FIGURE 4
Effect of WAY 121509 (100 mg/ml) on the interleukin-1β-induced NO synthesis in rat aortic rings during 48 h incubations under normal glucose conditions. Media was sampled and replaced at the end of 24 hr and sampled at the end of the next 24 hr (time point= 48 h). **Solid lines** represent periods during which rings were incubated in the presence of 0.05% DMSO. **Dashed lines** represent periods during which rings were incubated in the presence of WAY 121509 in 0.05% DMSO. Curve 1: rings treated with NGIL containing 0.05% DMSO during the first 24 h incubation period and replaced with identical treatment for the second 24 h. Curve 2: rings treated with NGIL containing 0.05% DMSO during the first 24 h period and replaced with NGIL containing 100 mg/ml WAY 121509 during the second 24 h. Curve 3: rings treated with NGIL containing 100 mg/ml WAY 121509 during the first 24 h incubation period and replaced with identical treatment for the second 24 h. Curve 4: rings treated with NGIL containing 100 mg/ml of WAY 121509 during the initial 24 h period only and replaced with media containing 0.05% DMSO during the last 24 h period. ANOVA repeated measures (Student-Newman-Keuls Method), p<0.001; *, incubation sequences (1, 2) versus (3, 4) at 24 h; and **, incubation sequences (1, 4) versus (2, 3) at 48 hr, p<0.05. N= 6 animals.

WAY 121509 exhibited significantly more NO synthesis at all time points compared to media without interleukin-1β and media containing WAY 121509. Aortic rings in high glucose media containing interleukin-1β had a 3-5 fold increase in nitrate/nitrite accumulation compared to normal glucose and interleukin-1β treatment at all time points studied. All groups incubated in interleukin-1β-free media showed no significant differences in NO production. In the presence of high glucose, WAY 121509 caused a significant inhibition (p<0.001) of interleukin-1β induction of NO synthesis. Similarly, WAY 121509 reduced interleukin-1β-induced NO synthesis in normal glucose media.

In the next study we compared the efficacy of WAY 121509 on blocking the effect of HGIL to its capability to reverse an already established effect of HGIL (Figure 3). To make this comparison, aortic rings were incubated in high glucose media containing interleukin-1β, with or without WAY 121509 present, during the first 24 hr, followed by GHIL with or without WAY 121509 during the second 24 hr. The vehicle, 0.05% DMSO was present when WAY 121509 was not. The presence of WAY 121509 during the first 24 h period and throughout the 48 h period caused an inhibitory effect on the interleukin-1β-stimulated NO synthesis. When aortic rings were incubated first in HGIL, the synthesis of NO continued to increase during the second 24 h. Replacement of HGIL media with HGILW after the first 24 h period resulted in a further 1.68-fold increase in nitrate/nitrite accumulation during the second 24 h period, this was not significantly different from NO levels measured for rings in HGILD throughout the 48 h. Aortic rings incubated in HGILW during the first 24 h and then incubated with HGILD during the second 24 h period demonstrated an increase in nitrite/nitrate accumulation. This was comparable to rings incubated in HGILD during both 24 h periods.

Rings incubated in the presence of interleukin-1β in normal media with or without WAY 121509 were studied (Figure 4). Aortic rings incubated with NGIL in the presence of WAY 121509 during the first 24 hr period and throughout the 48 hr period exhibited a significant reduction in nitrate/nitrite accumulation. Rings incubated in NGIL for the second 24 hr period with previous treatment in NGILW for the initial 24 hr period recovered the ability to synthesize NO to the same levels as rings in NGIL throughout 48 hr. Thus, the inhibitory effect of WAY 121509 applied before the induction of NO synthesis by interleukin-1β under high glucose conditions was not observed if WAY 121509 was applied after the induction of NOS had already occurred. Furthermore, the effect of WAY 121509 was reversible since its removal from the incubation media restored the interleukin-1β-induced NO synthesis to values similar to rings that had not been exposed at all the aldose reductase inhibitor.

### DISCUSSION

This is the first demonstration that an aldose reductase inhibitor (WAY 121509) antagonizes NO synthesis induced by a potent cytokine such as interleukin-1β. This antagonism was maintained for as long as WAY 121509 was present, regardless of whether the aortic rings were incubated in normal or high glucose conditions. Removal of WAY 121509 completely restored the NO synthesis to control levels, indicating its reversibility. Interestingly, in high glucose conditions, WAY 121509 was effective in inhibiting NO synthesis only when applied prior to NOS induction. Exposure of aortic rings to WAY 121509 after NOS induction effectively inhibited NO synthesis only under normal glucose conditions but failed under high glucose conditions, suggesting that high glucose-elicited changes in aortic rings suppressed the effect of WAY 121509 on NO synthesis.

These results in high glucose media showed a similar pattern to previous observations in diabetic and galactosemic (exaggerates the polyol pathway) animal models treated with aldose reductase inhibitors at different glycemic stages [28]. Administration of aldose reductase inhibitor at the onset of clinical diabetes [29] or experimental diabetes [25, 30] and galactosemia [3], results in the prevention of pathology-associated with excessive glucose metabolism by the polyol pathway probably due to the absence of chronic hyperglycemia. On the other hand, administration of aldose reductase inhibitors late in experimental diabetes or galactosemia was less effective at reducing pathology-associated with polyol pathway hyperactivity [28]. Thus, in both of these in vivo models and our in vitro high glucose model the early presence of WAY 121509 was necessary to block the hyperglycemic, diabetic, or galactosemic effect.

In experiments with rats and dogs, the efficacy of aldose reductase inhibitors at reducing polyol related pathology decreased as the condition progressed without intervention therapy [25]. In these studies of diabetic as well as galactosemic animal models the outcome of a particular trial depended on whether the aldose reductase inhibitor was administered at the onset, early or late in experimental diabetes or galactosemia.

WAY 121509 has been described as a highly specific aldose reductase inhibitor; however, explaining our observed results strictly on the basis of inhibition of an increase in polyol synthesis occurring only in the presence of high glucose is not straight forward. Aldose reductase inhibition is known to result in a decreased accumulation of intracellular sorbitol and in an increased availability of NADPH [31-34], a co-factor of NOS. Thus, NOS activity should increase, remain the same, but not decrease in the presence of WAY 121509, as is the case in our experiments. Furthermore, if WAY 121509 were blocking a high glucose-mediated increase in polyols, then one would expect it to block responses in high glucose but not in normal media. In fact, the opposite occurs: WAY 121509 inhibits previously induced NO synthesis in normal media but fails to inhibit the increased NO synthesis in high glucose media. An alternative explanation of the effect of WAY 121509 is that this aldose reductase inhibitor is acting in a non-specific manner [34, 35], inhibiting either the cytokine-elicited induction or the activity of the induced NOS, or both. The complete recovery of NO synthesis after removal of WAY 121509 might argue that only the activity of NOS was inhibited, but not its induction. On the other hand, WAY 121509 was not effective at reducing the activity of the already induced NOS. Additional experiments on iNOS enzyme activity, expression of the iNOS protein, or expression of iNOS mRNA might help identify the target of WAY 121509.

Even though the inhibition of interleukin-1β-induced NO by WAY 121509 is rather atypical, another explanation is that WAY 121509 might downregulate interleukin-1β-induced NO synthesis indirectly, but still as a consequence of inhibiting the polyol pathway. Lee et al. [36] observed in cultures of rat vascular smooth muscle, that the aldose reductase inhibitor zopolrestat could antagonize other interleukin-1β inducible events potentiated by high glucose concentrations such as prostaglandin production and cyclooxygenase (COX-2) expression. This study suggested that the mechanism by which high glucose increased interleukin-1β-induced COX-2 expression involved the indirect activation of protein kinase C through redox alteration elicited by the polyol pathway. Redox imbalances caused by the polyol pathway consists of an increased ratio of $NADP^+$/NADPH and NADH/$NAD^+$ due to increased aldose reductase and sorbitol dehydrogenase activities, respectively. As mentioned in the previous paragraph WAY 121509 should not have decreased interleukin-1β-induced NO synthesis based solely on the inhibition of the depletion of the NOS co-factor NADPH. Nevertheless, the impact of reducing the NADH/$NAD^+$ ratio downstream due to polyol inhibition might explain the inhibitory effect of WAY 121509 on interleukin-1β-induced NO synthesis. Elevation of the NADH/$NAD^+$ ratio via sorbitol accumulation results in the novo synthesis of diacylglycerol (DAG), which is associated with the activation of protein kinase C. Evidence supporting PKC activation in response to high glucose concentrations is well documented in a variety of cells and tissues including: retinal vascular endothelial, aortic endothelial and smooth muscle cells [37-39]; heart [40], isolated glomeruli [41], and in granulation tissue [42]. It is also well documented in vascular endothelial [43] and smooth muscle cells [39] that under hyperglycemic conditions cytokine-induced activation of the transcription factor NFkb increases possibly leading to up-regulation of interleukin-1β-induced NO synthesis [43, 44]. Thus, assuming that redox imbalance only takes place in tissues exposed to high glucose concentrations, then inhibiting increases in the NADH/NAD+ ratio through WAY 121509 could explain the observed reduction in interleukin-1β-induced NO synthesis. However, the fact that WAY 121509 inhibited partially interelukin-1β-induced NO synthesis at physiological levels of glucose suggests that the polyol pathway is active under this conditions. Indeed, it has been reported that even under normoglycemic conditions aldose reductase inhibitors reduce the flux of glucose via the sorbitol pathway effectively decreasing both synthesis of polyols and NADH/NAD+ ratio [45].

In conclusion, high glucose up-regulation of interleukin-1β-induced NO synthesis might be linked to redox imbalances caused by the polyol pathway. Therefore, increased synthesis of cytokine-induced NO through high glucose evoked redox imbalance could be a relevant pathophysiological mechanism underlying vascular pathogenesis in diabetes.

### REFERENCES

1. Tomlinson, D.R., Willars, G.B. and Carrington, A.L. (1992) Aldose reductase inhibitors and diabetic complications, *Pharmacol. Ther.,* **54**, 151-194.
2. Gonzalez, R.G, Barnett, P., Aguayo, J., Cheng, H.M. and Chylack, L.T. (1984) Direct measurement of polyol pathway activity in the ocular lens, *Diabetes,* **33**, 196-199.
3. Frank, R.N. (1995) The galactosemic dog. A valid model for both early and late stages of diabetic retinopathy [editorial; comment], *Arch. Ophthalmol.,* **113**, 275-276.
4. Okuda, Y., Bannai, C., Nagahama, M., Mizutani, M., Mitsui, Y. and Yamashita, K. (1991) Effect of glucose and an aldose reductase inhibitor on myo-inositol uptake by cultured human endothelial cells, *Diabetes Res.,* **18**, 61-64.
5. Obrosova, I., Faller, A., Burgan, J., Ostrow, E. and Williamson, J.R. (1997) Glycolytic pathway, redox state of NAD(P)-couples and energy metabolism in lens in galactose-fed rats: effect of an aldose reductase inhibitor, *Curr. Eye Res.,* **16**, 34-43.
6. Ruderman, N.B., Williamson, J.R. and Brownlee, M. (1992) Glucose and diabetic vascular disease, *FASEB J.*, **6**, 2905-2914.
7. Williamson, J.R., Ostrow, E., Eades, D., Chang, K., Allison, W., Kilo, C. and Sherman, W.R. (1990) Glucose-induced microvascular functional changes in non-diabetic rats are stereospecific and are prevented by an aldose reductase inhibitor, *J. Clin. Invest.,* **85**, 1167-1172.
8. Martinez-Nieves, B. and Dunbar, J.C. (2001) The effect of diabetes and sex on nitric oxide-mediated cardiovascular dynamics, *Exp. Biol. Med. (Maywood),* **226**, 37-42.
9. Rodriguez-Manas, L., Angulo, J., Peiro, C., Llergo, J.L. and Sanchez-Ferrer, A. (1998) Endothelial dysfunction and metabolic control in streptozotocin-induced diabetic rats, *Br. J. Pharmacol.,* **123**, 1495-1502.
10. Wu, G. and Meininger, C.J. (1995) Impaired arginine metabolism and NO synthesis in coronary endothelial cells of the spontaneously diabetic BB rat, *Am. J. Physiol.,* **269**, H1312-H1318.
11. Dunlop, M. (2000) Aldose reductase and the role of the polyol pathway in diabetic nephropathy, *Kidney Int.,* **58**, S3-12.
12. Tesfamariam, B., Gupta, S., Oates, P.J., Ruderman, N.B. and Cohen, R.A. (1993) Aldose reductase inhibition restores endothelial cell function in diabetic rabbit aorta, *J. Cardiovasc. Pharmacol.,* **21**, 205-211.
13. Trueblood, N. and Ramasamy, R. (1998) Aldose reductase inhibition improves altered glucose metabolism of isolated diabetic rat hearts, *Am. J. Physiol.,* **275**, H75-H83.
14. Yoshida, M., Sugiyama, Y., Akaike, N., Ashizawa, N., Aotsoka, T. and Ohibayashi, S. (1998) Amelioration of neurovascular deficits in diabetic rats by a novel aldose reductase inhibitor, GP-1447: minor contribution of nitric oxide, *Diabetes Res. Clin. Pract.,* **40**, 101-112.
15. Morrison, A.D. (1990) Effect of inhibition of polyol pathway activity on aortic smooth muscle metabolism, *Clin. Invest. Med.,* **13**: 119-122.
16. Cameron, N. and Cotter, M.A. (1993) Contraction and relaxation of aortas from galactosaemic rats and the effects of aldose reductase inhibition, *Eur. J. Pharmacol.,* **243**, 47-53.
17. Sakakibara, F., Hotta, N., Koh, N. and Sakamoto, N. (1993) Effects of high glucose concentrations and epalrestat on sorbitol and myo-inositol metabolism in cultured rabbit aortic smooth muscle cells, *Diabetes* **42**, 1594-1600.
18. Taylor, P.D., Wickenden, A.D., Mirrlees, D.J. and Poston, L. (1994) Endothelial function in the isolated perfused mesentery and aortae of rats with streptozotocin-induced diabetes: effect of treatment with the aldose reductase inhibitor, ponalrestat, *Br. J. Pharmacol.,* **111**, 42-48.
19. Arauz-Pacheco, C., Ramirez, L.C., Pruneda, L., Sanborn, G.E., Rosenstock, J. and Raskin, P. (1992) The effect of the aldose reductase inhibitor, ponalrestat, on the progression of diabetic retinopathy, *J. Diabetes Complications,* **6**, 131-137.
20. Cotter, M.A., Cameron, N.E. and Hohman, T.C. (1998) Correction of nerve conduction and endoneurial blood flow deficits by the aldose reductase inhibitor, tolrestat, in diabetic rats, *J. Peripher. Nerv. Syst.* **3**, 217-223.
21. Kato, N., Mizuno, K., Makino, M., Suzuki, T. and Yagihashi, S. (2000) Effects of 15-month aldose reductase inhibition with fidarestat on the experimental diabetic neuropathy in rats [In Process Citation], *Diabetes Res. Clin. Pract.* **50**, 77-85.

22. Tilton, R.G., Pugliese, G., LaRose, L.S., Faller, A.M., Chang, K., Province, M.A. and Williamson, J.R. (1991) Discordant effects of the aldose reductase inhibitor, sorbinil, on vascular structure and function in chronically diabetic and galactosemic rats, *J. Diabet. Complications,* **5**, 230-237.

23. Stevens, M.J., Dananberg, J., Feldman, E.L., Lattimer, S.A., Kamijo, M., Thomas, T.P., Shindo, H., Sima, A.A. and Greene, D.A. (1994) The linked roles of nitric oxide, aldose reductase and, ($Na^+$,$K^+$)-ATPase in the slowing of nerve conduction in the streptozotocin diabetic rat, *J. Clin. Invest.*, **94**, 853-859.

24. Dvornik, D., Hohman, T.C. and Basso, M.D. (1996) Aminoguanidine does not inhibit aldose reductase activity in galactose- fed rats, *J. Diabetes Complications,* **10**, 23-30.

25. Robinson, W.G., Jr., Laver, N.M., Jacot, J.L., Glover, J.P., Basso, M.D., Blouin, P. and Hohman, T.C. (1996) Diabetic-like retinopathy ameliorated with the aldose reductase inhibitor WAY-121,509, *Invest. Ophthalmol. Vis. Sci.,* **37**, 1149-1156.

26. Cameron, N.E., Cotter, M.A., Basso, M. and Hohman, T.C. (1997) Comparison of the effects of inhibitors of aldose reductase and sorbitol dehydrogenase on neurovascular function, nerve conduction and tissue polyol pathway metabolites in streptozotocin-diabetic rats, *Diabetologia,* **40**, 271-281.

27. Keegan, A., Jack, A.M., Cotter, M.A. and Cameron, N.E. (2000) Effects of aldose reductase inhibition on responses of the corpus cavernosum and mesenteric vascular bed of diabetic rats, *J. Cardiovasc. Pharmacol.,* **35**, 606-613.

28. Engerman, R.L. and Kern, T.S. (1993) Aldose reductase inhibition fails to prevent retinopathy in diabetic and galactosemic dogs [see comments], *Diabetes,* **42**, 820-825.

29. Judzewitsch, R.G., Jaspan, J.B., Polonsky, K.S., Weinberg, C.R., Halter, J.B., Halar, E., Pfeifer, M.A., Vukadinovic, C., Bernstein, L., Schneider, M., Liang, K.Y., Gabbay, K.H., Rubenstein, A.H. and Porte, D. (1983) Aldose reductase inhibition improves nerve conduction velocity in diabetic patients, *N. Engl. J. Med.,* **308**, 119-125.

30. Chakrabarti, S. and Sima, A.A. (1989) Effect of aldose reductase inhibition and insulin treatment on retinal capillary basement membrane thickening in BB rats, *Diabetes,* **38**, 1181-1186.

31. Cameron, N.E. and Cotter, M.A. (1993) Potential therapeutic approaches to the treatment or prevention of diabetic neuropathy: evidence from experimental studies, *Diabet. Med.,* **10**, 593-605.

32. Greene, D.A., Sima, A.A., Stevens, M.J., Feldman, E.L., Killen, P.D., Henry, D.N., Thomas, T., Dananberg, J. and Lattimer, S.A. (1993) Aldose reductase inhibitors: an approach to the treatment of diabetic nerve damage, *Diabetes Metab. Rev.,* **9**, 189-217.

33. Stevens, M.J., Lattimer, S.A., Kamijo, M., Van Huysen, C., Sima, A.A. and Greene, D.A. (1993) Osmotically-induced nerve taurine depletion and the compatible osmolyte hypothesis in experimental diabetic neuropathy in the rat, *Diabetologia,* **36**, 608-614.

34. Kapor-Drezgic, J., Zhou, X., Babazono, T., Dlugosz, J.A., Hohman, T. and Whiteside, C. (1999) Effect of high glucose on mesangial cell protein kinase C-delta and - epsilon is polyol pathway-dependent, *J. Am. Soc. Nephrol.,* **10**, 1193-1203.

35. Sellers, D.J. and Chess-Williams, R. (2000) The effects of streptozotocin-induced diabetes and aldose reductase inhibition with sorbinil, on left and right atrial function in the rat [In Process Citation], *J. Pharm. Pharmacol.,* **52**, 687-694.

36. Lee, S.H., Woo, H.G., Baik, E.J. and moon, C.H. (2000) High glucose enhances IL-beta-induced cyclooxygenase-2 expression in rat vascular smooth muscle cells, *Life Sci.,* **68**, 57-67.

37. Lee, T.S., Saltsman, K.A., Ohashi, H. and King, G.L. (1989) Activation of protein kinase C by elevation of glucose concentration: proposal for a mechanism in the development of diabetic vascular complications, *Proc. Natl. Acad. Sci. USA,* **86**, 5141-5145.

38. Williams, B. and Schrier, R.W. (1992) Characterization of glucose-induced in situ protein kinase C activity in cultured vascular smooth muscle cells, *Diabetes,* **41**, 1464-1472.

39. Yerneni, K.K., Bai, W., Khan, B.V., Medford, R.M. and Natarajan, R. (1999) Hyperglycemia-induced activation of nuclear transcription factor kappaB in vascular smooth muscle cells, *Diabetes,* **48**, 855-864.

40. Okumura, K., Akiyama, N., Hashimoto, H., Ogawa, K. and Satake, T. (1988) Alteration of 1,2-diacylglycerol content in myocardium from diabetic rats, *Diabetes,* **37**, 1168-1172.

41. Craven, P.A., Davidson, C.M. and DeRubertis, F.R. (1990) Increase in diacylglycerol mass in isolated glomeruli by glucose from de novo synthesis of glycerolipids, *Diabetes,* **39**, 667-674.

42. Wolf, B.A., Williamson, J.R., Easom, R.A., Chang, K., Sherman, W.R. and Turk, J. (1991) Diacylglycerol accumulation and microvascular abnormalities induced by elevated glucose levels, *J. Clin. Invest.,* **87**, 31-38.

43. Suschek, C., Fehsel, K., Kroncke, K.D., Sommer, A. and Kolb-Bachofen, V. (1994) Primary cultures of rat islet capillary endothelial cells. Constitutive and cytokine-inducible macrophage like nitric oxide synthases are expressed and activities regulated by glucose concentration, *Am. J. Pathol.,* **145**, 685-695.

44. Sharma, K., Danoff, T.M., DePiero, A. and Ziyadeh, F.N. (1995) Enhanced expression of inducible nitric oxide synthase in murine macrophages and glomerular mesangial cells by elevated glucose levels: possible mediation via protein kinase C, *Biochem. Biophys. Res. Commun.,* **207**, 80-88.

45. Pugliese, G., Tilton, R.G. and Williamson, J.R. (1991) Glucose-induced metabolic imbalances in the pathogenesis of diabetic vascular disease, *Diabetes Metab. Rev.,* **7**, 35-59.

